# ADENOCARCINOMA AND DYSPLASIA IN BARRETT ESOPHAGUS: CRITICAL ANALYSIS OF RISK FACTORS AND SURVEILLANCE PROTOCOLS

**DOI:** 10.1590/0102-6720202400033e1826

**Published:** 2024-11-15

**Authors:** Eduardo GALLON, Sérgio SZACHNOWICZ, André Fonseca DUARTE, Francisco TUSTUMI, Rubens Antonio Aissar SALLUM, Paulo HERMAN, Ulysses RIBEIRO

**Affiliations:** 1 Universidade de São Paulo, Faculty of Medicine, Department of Gastroenterology - São Paulo (SP), Brazil

**Keywords:** Barrett Esophagus, Gastroesophageal Reflux Diseases, Adenocarcinoma, Epidemiology., Esôfago de Barrett, Refluxo Gastroesofágico, Adenocarcinoma, Epidemiologia.

## Abstract

**BACKGROUND:**

Identification of epidemiological risk factors in Barrett esophagus resulting in dysplasia and adenocarcinoma and its impact on prevention and early detection.

**AIMS:**

To evaluate epidemiological risk factors involved in the development of dysplasia and esophageal adenocarcinoma from Barrett esophagus in a specific population. To critically analyze the surveillance period, aiming to individualize follow-up time according to identified risks.

**METHODS:**

A retrospective case-control study was carried out at a tertiary center involving patients diagnosed and followed up for Barrett esophagus. Patients who developed esophageal adenocarcinoma and/or dysplasia were compared to those who did not, considering variables such as gender, age, smoking status, body mass index, ethnicity, and Barrett esophagus extension. Logistic regression was performed to measure the odds ratio for risk factors associated with the outcome of adenocarcinoma and dysplasia. The presence of epidemiological risk factors in this population was correlated with the time taken to develop esophageal adenocarcinoma from metaplasia.

**RESULTS:**

A statistically significant difference was observed in smoking status, race, gender, Barrett esophagus extension, and age between the group with esophageal adenocarcinoma and the group without it. Smokers and former smokers had a 4.309 times higher risk of developing esophageal adenocarcinoma, and each additional centimeter of Barrett esophagus increased the risk by 1.193 times. In the dysplasia group, smoking status, Barrett esophagus extension, and age were statistically significant factors; each additional centimeter of Barrett esophagus extension increased the risk of dysplasia by 1.128 times, and each additional year of age increased the risk by 1.023 times. Patients without risk factors did not develop esophageal adenocarcinoma within 12 months, even with prior dysplasia.

**CONCLUSIONS:**

The study confirmed a higher risk of developing dysplasia and esophageal adenocarcinoma in specific epidemiological groups, allowing for more cost-effective monitorization for patients with Barrett esophagus.

## RESUMO

**RACIONAL:**

Identificação de fatores de risco epidemiológicos no esôfago de Barrett resultando em displasia e adenocarcinoma e seu impacto na prevenção e detecção precoce.

**OBJETIVOS:**

Avaliar fatores de risco epidemiológicos envolvidos no desenvolvimento de displasia e adenocarcinoma a partir do Barrett em população específica. Realizar análise crítica do período de vigilância, objetivando individualizar o tempo de seguimento conforme riscos identificados.

**MÉTODOS:**

Estudo caso-controle retrospectivo em centro terciário com pacientes com esôfago de Barrett diagnosticados e seguidos neste centro. Pacientes com Barrett que apresentaram adenocarcinoma e/ou displasia foram comparados aos que não apresentaram, levando em consideração as variáveis sexo, idade, tabagismo, IMC, etnia e extensão do Barrett. Posteriormente, foi realizada regressão logística para mensuração da razão de chances entre fatores de risco para o desfecho adenocarcinoma e desfecho displasia. Foi correlacionada a presença de fatores epidemiológicos de risco nessa população com o tempo de desenvolvimento de adenocarcinoma a partir da metaplasia.

**RESULTADOS:**

Houve diferença estatisticamente significante entre as variáveis tabagismo, raça, sexo, extensão do Barrett e idade no grupo com adenocarcinoma em relação ao sem adenocarcinoma; tabagistas e ex-tabagistas apresentaram risco 4,309 vezes maior de desenvolver adenocarcinoma; a extensão do Barrett aumentou o risco em 1,193 vezes a cada centímetro. No grupo com displasia, as variáveis tabagismo, extensão do Barrett e idade se mostraram significantes estatisticamente; extensão do Barrett aumentou 1,128 vezes a cada centímetro o risco de displasia e idade aumentou 1,023 a cada ano o risco desse desfecho. Pacientes sem fatores de risco não desenvolveram adenocarcinoma em menos de 12 meses, mesmo com displasia anteriormente.

**CONCLUSÕES:**

O estudo confirmou maior risco de desenvolver displasia e adenocarcinoma em grupos epidemiológicos específicos, podendo direcionar o seguimento em pacientes com Esôfago de Barrett de forma mais custo efetiva.

## INTRODUCTION

Gastroesophageal reflux disease (GERD) is caused by the involuntary and repetitive return of gastric contents into the esophagus^11^. Due to the progressive damage from refluxate, a natural repair process of the esophageal mucosa can occur, resulting in the replacement of squamous epithelium with glandular columnar epithelium, accompanied by the appearance of goblet cells typical of intestinal mucosa. This process characterizes intestinal metaplasia, known as Barrett Esophagus (BE)^28^.

The incidence of GERD in Brazil is estimated at 12%, which corresponds to approximately 24 million individuals. However, the actual prevalence of this disease may be even higher, as many individuals access clinical treatment informally^19^. BE occurs in 10 to 15% of patients with GERD, especially in those with a long history of reflux^11^. Thus, BE affects approximately 3 million people in Brazil, with an increase in its incidence observed in recent years due to changes in the population’s dietary patterns and aging^11^.

For the definitive diagnosis of BE, upper digestive endoscopy associated with biopsy is necessary to confirm the histopathological presence of goblet cells in the esophageal epithelium^3,5,18^.

Currently, the follow-up of BE aims at the early detection of dysplasia and esophageal adenocarcinoma (EAC). This follow-up is standardized by high-definition white-light endoscopy, with random four-quadrant biopsies every 2 cm (or every 1 cm if dysplasia is known or suspected), according to the Seattle protocol^7^. Additionally, biopsies are performed in areas of mucosal irregularities such as nodules, ulcers, or visible lesions^22^. Even in patients who have undergone surgical and endoscopic treatment for BE, the development of EAC should be monitored in a standardized manner^4^.

The description of Barrett epithelium should follow the Prague classification, which considers the circumferential extent (C) and the maximum extent (M) of the columnar epithelium with metaplasia^24^.

Several risk factors have been identified for the development of BE. Endoscopic database studies have reported that the prevalence of BE increases sharply in the fourth and fifth decades of life. Male gender, white/Caucasian race, chronic symptomatic reflux (symptoms more than once a week for more than 5 years), and central obesity (measured by waist-to-hip ratio or waist circumference) are other associated risk factors^26^.

BE presents a genetically unstable epithelium with risks for dysplasia and EAC. Patients with BE have an 11-fold higher relative risk of developing EAC, which may result from a metaplasia-dysplasia-carcinoma sequence^18^. The annual risk of esophageal adenocarcinoma from BE without dysplasia is 0.12% (95%CI, 0.09 to 0.15)^12^. In our setting, the current prevalence of EAC development in patients under follow-up is 0.19%^27^.

Dysplasia, characterized by abnormal organization or disordered differentiation of cells or tissue in an organ, is considered a premalignant lesion. Dysplasia remains the primary biological predictor for progression to esophageal adenocarcinoma, despite active research into biological and molecular markers. Efforts should focus on the early detection of dysplasia and EAC in BE to avoid diagnosing adenocarcinoma at an advanced stage, which leads to a worse prognosis, with an overall five-year survival rate of approximately 18%^17^.

In a meta-analysis conducted by Krishnamoorthi, including 74,943 patients, the epidemiological factors involved in the progression of BE to adenocarcinoma were evaluated. The analysis found that age (OR 1.47, 95%CI 1.01-1.05), male gender (OR 2.16, 95%CI 1.84-2.53), smoking (OR 1.47, 95%CI 1.09-1.98), and BE segment length (OR 1.25, 95%CI 1.16-1.36) were significant factors^15^.

However, despite the presence of epidemiological risk factors for the progression of BE to EAC, the follow-up recommendations for BE proposed by different international societies (Table 1) considered only the presence or absence of dysplasia and its grade as variables to define the follow-up intervals, without taking into account known epidemiological factors in the literature.

**Table 1:** International Society Guidelines for Barrett Esophagus Surveillance.

-	AGA^1^	ACG^23^	ASGE^2^	BSG^6,10^	ESGE^29^	Australian^30^
BEWD	Surveillance every 3-5 years	Surveillance every 3-5 years	Surveillance every 3-5 years	Surveillance every 3-5 years. If length <3 cm without intestinal metaplasia/dysplasia → repeat EGD. If repeat EGD is negative → discontinue surveillance. If repeat EGD is positive for intestinal metaplasia → surveillance every 3-5 years. If length is ≥3 cm → surveillance every 2-3 years.	<1 cm → no surveillance ≥1 and <3 cm: surveillance every 5 years ≥3 and <10 cm: surveillance every 3 years ≥10 cm: consult a BE specialist center. Continue surveillance until at least 75 years of age.	Short segment (<3 cm): repeat EGD in 3-5 years. Long segment (⩾3 cm): repeat EGD in 2-3 years.
BE with IND	No information	Repeat EGD after PPI therapy for 3-6 months. If the repeat EGD shows indefinite, then surveillance every 12 months	Additional pathological review, increased dose of PPI therapy, and repeat EGD with biopsy	Repeat EGD after PPI therapy for 6 months. If it indicates BE without dysplasia again, follow this BEWD protocol.	Repeat EGD after PPI therapy for 6 months. If it shows IND or BEWD, follow the BEWD protocol.	Repeat EGD after PPI therapy for 6 months. If it shows BEWD/LGD/HGD/EAC, follow the respective protocols. If it shows IND, repeat EGD in 6 months.
BE with LGD	Surveillance every 6-12 months	Endoscopic eradication therapy is recommended for confirmed LGD without life-limiting comorbidities or surveillance every 12 months	Repeat EGD in 6 months to confirm the diagnosis. Subsequently, perform annual surveillance EGD in selected patients	Repeat EGD in 6 months. If LGD persists, EET is recommended or surveillance every 6 months.	Repeat EGD in 6 months. If it shows complete eradication of dysplasia (BEWD), then perform EGD annually until two consecutive results show BEWD. Then, follow the BEWD protocol. If LGD persists, then EET is recommended	Repeat EGD every 6 months until two consecutive results show BEWD. Then, follow a less frequent surveillance schedule.
BE with HGD	EET or surveillance every 3 months	EET for HGD confirmed without life-limiting comorbidities	EET or surveillance every 3 months	EET.	EET. If biopsies show BEWD repeat EGD in 3 months.	EET or surveillance every 3 months.

AGA: American Gastroenterological Association; ACG: American College of Gastroenterology; ASGE: American Society for Gastrointestinal Endoscopy; BSG: British Society of Gastroenterology; ESGE: European Society of Gastrointestinal Endoscopy; BEWD: Barrett Esophagus without Dysplasia; EGD: Esophagogastroduodenoscopy; BE: Barrett esophagus; IND: Indefinite Dysplasia; PPI: Proton-pump inhibitor; LGD: Low-Grade Dysplasia; HGD: High-Grade Dysplasia; EAC: esophageal adenocarcinoma; EET: Endoscopic eradication therapy.

It is necessary to better understand the epidemiological factors associated with the development of dysplasia or EAC in BE in our population and to critically analyze the proposed intervals among international societies to promote personalized screening. This was the motivation behind conducting the present study.

The objective of this study was to evaluate the relationship of epidemiological risk factors for the development of dysplasia/adenocarcinoma from BE in a specific population, as well as to conduct a critical analysis of the BE follow-up period, with the aim of individualizing the follow-up time according to the identified risk factors.

## METHODS

### Study design

A retrospective case-control study was conducted within the Esophageal Surgery Service at the University Hospital of the School of Medicine of Universidade de São Paulo, collecting data from patients with BE followed by this service. The study was approved by the Ethics Committee of the Institution.

### Eligibility criteria

Patients diagnosed with BE via endoscopy at the service and who were subsequently followed up at this center were included in the analysis, totaling 646 patients attended between 1991 and 2020. Among these, 71 presented with dysplasia and 21 presented with EAC.

### Variables studied

• Gender (male and female)
• Age
• Smoking status (current smoker, former smoker, and non-smoker)
• Body Mass Index (BMI)
• Ethnicity (Asian, white, mixed-race, and black)
• Extent of BE

### Studied outcome

The outcomes studied were EAC and dysplasia in patients diagnosed with BE. Patients with confirmed BE, as determined by endoscopy performed at the service, were divided into two groups: those who presented with EAC or dysplasia and those who did not. The incidence of variables was compared between these groups.

The variables gender, smoking status, and ethnicity were compared with the presence or absence of dysplasia and EAC using the ꭓ^2^ test. The variables extent of BE, BMI, and age were compared between the two groups using the Student’s *t*-test. A significance level (alpha) of 5% was adopted.

**Figure 1:**
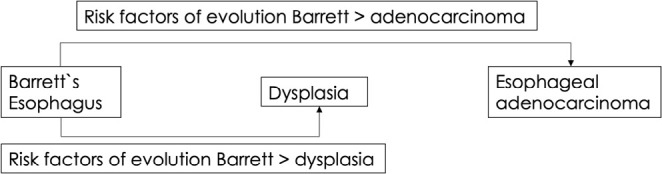
Flowchart of the progression of Barrett esophagus to esophageal adenocarcinoma and dysplasia

Variables that demonstrated a significant difference (p<0.05) in the progression of BE to EAC were selected, and the odds ratio (OR) for these outcomes was measured through logistic regression, as shown in the diagram below (Figure 1). During this stage, the ethnicity variable was categorized as white and non-white, and the smoking status group was divided into smoker or former smoker and non-smoker. A significance level (alpha) of 5% and a 95% confidence interval were considered. The same process was applied to variables that showed a significant difference in the progression of BE to dysplasia.

The evaluation of risk factors for patients who progressed from esophageal dysplasia to EAC was not performed because all patients with EAC, for whom variable information was available, had previously presented with dysplasia.

Finally, an intragroup analysis was conducted on the follow-up endoscopies of patients who developed dysplasia and/or EAC. The maximum endoscopy interval capable of identifying 100% of the sought outcomes was analyzed and compared between patients with and without the risk factors identified by logistic regression.

## RESULTS

Table 2 shows the relationship between qualitative variables (gender, smoking status, and ethnicity) and the incidence of EAC. A statistically significant difference was found among different ethnicities (p=0.027), among smokers, non-smokers, and former smokers (p<0.001), and between different genders (p=0.019).

**Table 2:** Comparison of qualitative variables between groups with and without esophageal adenocarcinoma.

	Smoker			Ethnicity				Gender	
	Yes	No	Former	Asian	White	Mixed-race	Black	Male	Female
Esophageal adenocarcinoma	8	5	4	2	15	2	0	17	4
No esophageal adenocarcinoma	52	221	36	7	409	28	14	345	280
p-value	p<0.001			p=0.027				p=0.019	

Table 3 shows the relationship between qualitative variables and the incidence of dysplasia. A statistically significant difference was observed in dysplasia incidence among smoking statuses (p=0.04). However, there was no significant difference in dysplasia incidence between different genders (p=0.155) and ethnicities (p=0.325).

**Table 3:** Comparison of qualitative variables between groups with and without dysplasia.

	Smoker			Ethnicity				Gender	
	Yes	No	Former	Asian	White	Mixed-race	Black	Male	Female
Dysplasia	17	33	6	3	58	5	3	46	25
No dysplasia	43	193	34	6	366	25	11	316	259
p-value	p=0.04			p=0.325				p=0.155	

Table 4 displays the relation between quantitative variables (extent of BE, BMI, and age) and the incidence of EAC. Using the Student’s *t*-test, significant differences were observed for the extent of BE (p=0.002) and age (p=0.044), while BMI (p=0.449) did not show a statistically significant difference.

**Table 4:** Comparison of quantitative variables between groups with and without esophageal adenocarcinoma.

	Extent of Barrett esophagus				Body Mass Index				Age			
	Mean	SD	Max	Min	Mean	SD	Max	Min	Mean	SD	Max	Min
Esophageal adenocarcinoma	5.325	3.221	12	1	26.600	4.913	36.707	18.326	62.000	14.843	88	29
No esophageal adenocarcinoma	3.296	2.859	18	0.3	27.706	5.161	53.250	11.157	55.256	14.733	92	14
p-value	p=0.002				p=0.449				p=0.044			

SD: standard deviation; Max: Maximum; Min: Minimum.

Table 5 outlines the relationship between quantitative variables and the incidence of dysplasia. Using the Student’s *t*-test, significant differences were observed for the extent of BE (p=0.004) and age (p=0.049), while BMI (p=0.240) did not show a statistically significant difference.

**Table 5:** Comparison of quantitative variables between groups with and without dysplasia.

	Extent of Barrett esophagus				Body Mass Index				Age			
	Mean	SD	Max	Min	Mean	SD	Max	Min	Mean	SD	Max	Min
Dysplasia	4.294	3.139	14	0.5	26.925	5.066	41.913	18.326	58.743	13.918	88	22
No dysplasia	3.243	2.839	18	0.3	27.814	5.161	53.250	11.157	55.063	14.835	92	14
p-value	p=0.004				p=0.240				p=0.049			

SD: standard deviation; Max: Maximum; Min: Minimum.

The significant variables for the development of EAC were smoking status, ethnicity, gender, extent of BE, and age. While the significant variables for the development of dysplasia were smoking status, extent of BE, and age.

In the logistic regression analysis (Table 6), smokers or former smokers exhibited an odds ratio of 4,309 times higher for developing EAC from BE (p=0.014). Regarding the extent of BE, each centimeter increase was associated with an odds ratio of 1,193 for developing EAC (p=0.017).

**Table 6:** Logistic regression for the outcome of esophageal adenocarcinoma.

	Odds ratio	p-value
Intercept	0.001	<0.001
Ethnicity (not white)	2.079	0.266
Gender (male)	1.834	0.387
Age	1.029	0.163
Extent of Barrett esophagus	1.193	0.017
Smoker (Yes or Former)	4.309	0.014
Dysplasia	206.020	<0.001

In the analyzed sample, patients with dysplasia had an odds ratio 206 times higher for developing EAC compared to those without dysplasia (Table 6). This finding aligns with the literature, which identifies dysplasia as the primary marker for progression to EAC.

**Figure 2:**
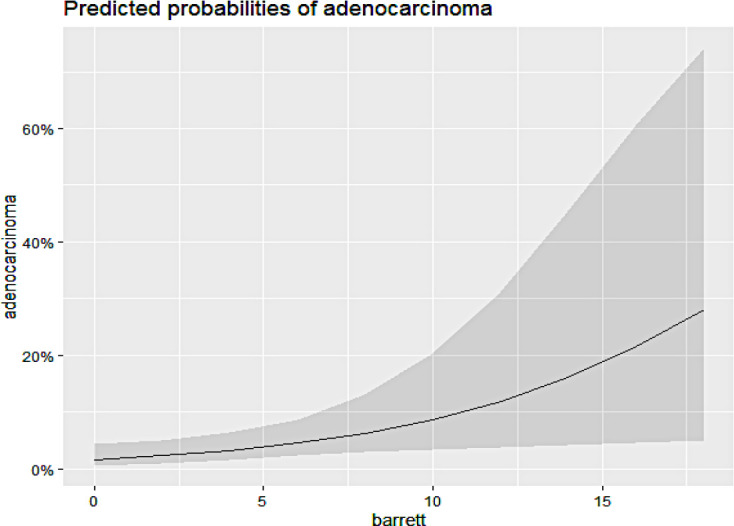
Relationship between adenocarcinoma and the extent of Barrett esophagus

**Figure 3:**
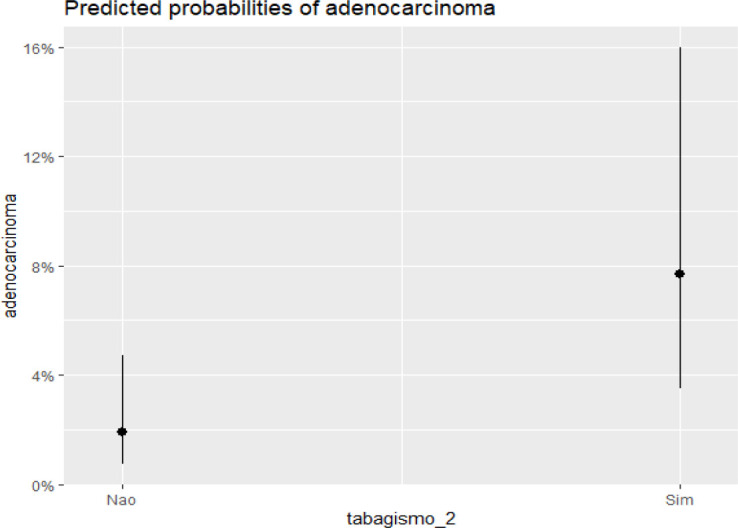
Relationship between esophageal adenocarcinoma and smoking status.

Figure 2 illustrates that an increase in the extent of BE corresponds with a higher incidence of EAC. Similarly, Figure 3 shows a higher incidence of EAC among smokers and former smokers.

In the logistic regression analysis to identify the odds ratio for the development of dysplasia from BE, shown in Table 7, age was associated with an odds ratio of 1,023 times higher for developing dysplasia with each additional year of life (p=0.041). The extent of BE was identified as a significant factor, increasing the chance of dysplasia by 1,128 times with each centimeter (p=0.008).

**Table 7:** Logistic regression for the outcome of dysplasia.

	Odds ratio	p-value
Intercept	0.030	<0.001
Smoker (Yes or Former)	1.538	0.167
Extent of Barrett esophagus	1.128	0.008
Age	1.023	0.041

**Figure 4:**
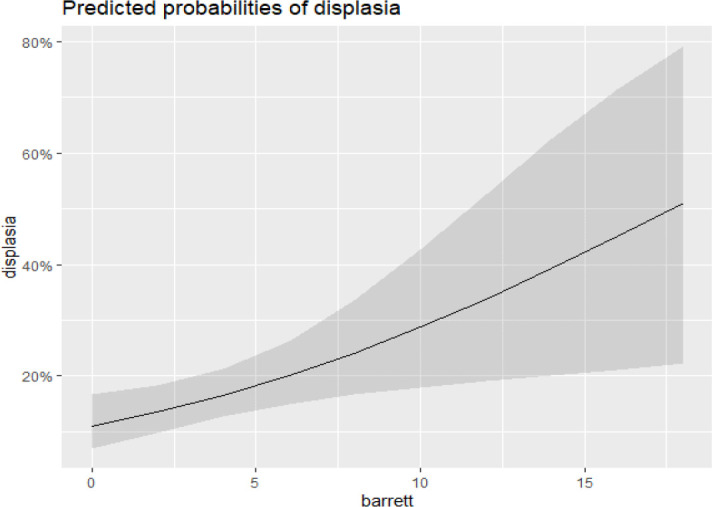
Relationship between dysplasia and the extent of Barrett esophagus.

**Figure 5:**
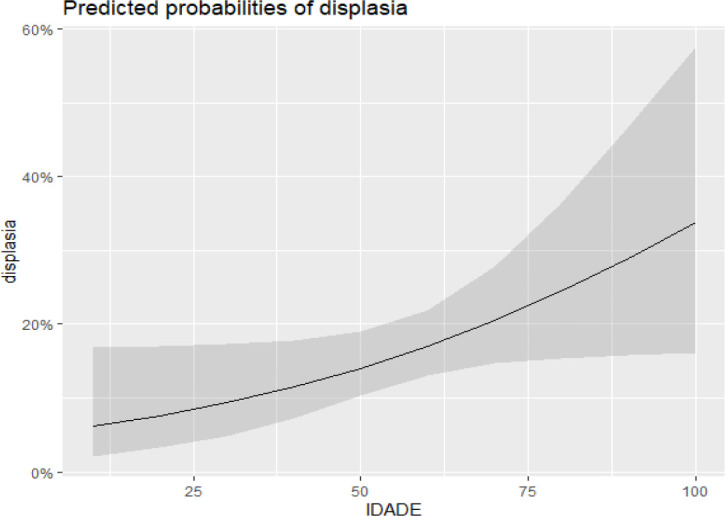
Relationship between dysplasia and age.

Figure 4 demonstrates that an increase in the extent of BE is associated with a higher incidence of dysplasia. Similarly, Figure 5 shows that the incidence of dysplasia increases with age.

Considering the period in which smokers or former smokers developed EAC from BE (noting that all previously presented with dysplasia), it was observed that if follow-up were conducted every 6 months, 100% of patients would receive an early diagnosis of EAC. On the other hand, for non-smokers, 100% of the diagnoses would be made early if follow-up were conducted every 12 months. Conversely, if smokers were followed up every 12 months, only 81.81% of cases would be diagnosed early.

For patients with long-segment BE who developed EAC from BE (noting that all had previously presented with dysplasia), a 6-month follow-up would result in a 100% early diagnosis rate. For patients with short-segment BE, a 12-month follow-up would ensure a 100% early diagnosis rate. However, if long-segment BE patients were followed every 12 months, only 75% of cases would be diagnosed early.

In patients aged ≥60 who developed dysplasia from BE, a 6-month follow-up interval would result in a 100% early diagnosis rate for EAC. For patients under 60 years old, 100% of the diagnoses would be made early if the follow-up were conducted every 12 months. However, if patients aged ≥60 were followed every 12 months, only 92.5% of cases would be diagnosed early.

No significant difference was observed in the early detection of dysplasia cases between patients with short-segment and long-segment BE. Thus, a 6-month follow-up interval would result in a 100% early diagnosis rate in both groups, while a 12-month follow-up would reduce the detection rate by approximately 7% in both groups.

**Figure 6:**
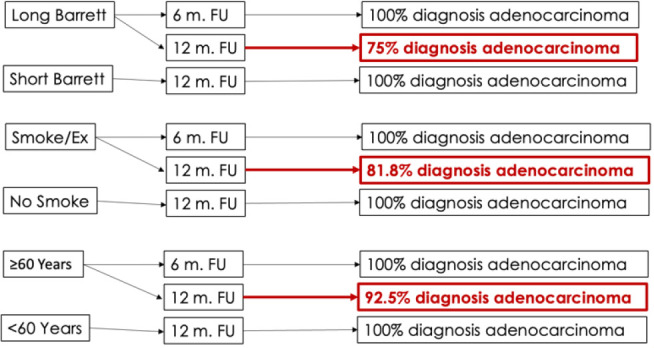
Relationship between the follow-up time of individuals with risk factors identified in logistic regression and the percentage of early esophageal adenocarcinoma diagnosis.

Legend: m: months; FU: follow-up; Ex: former smoker.

These results are illustrated in Figure 6.

## DISCUSSION

Given that most EACs arise in the presence of BE, it is necessary to establish a well-defined surveillance system, as early detection of BE through endoscopic surveillance allows for identifying the progression to dysplasia or EAC in its early stages, facilitating endoscopic or surgical treatment with higher success rates^14^. This scenario was demonstrated in a cohort study of 30,000 patients with BE followed for 5 years, where patients diagnosed with EAC during surveillance were detected at an earlier stage (stage 0 to 1: 74.7 *versus* 56.2%; p<0.001), survived longer (median 3.2 *versus* 2.3 years; p<0.001), and had lower cancer-related mortality (34.0 *versus* 54.0%; p<0.0001) compared to those not under surveillance^8^.

However, the approach to follow-up and surveillance of BE remains controversial in the literature and across various international guidelines for BE, as presented in the introduction of this study. Considering the conditions and limitations of the Brazilian public health system, along with the difficulty in increasing the number of endoscopic exams for screening^21^, it is imperative to conduct a detailed study on the risk factors for developing dysplasia and EAC that can be practically evaluated in the Brazilian population. This aims to adapt the surveillance criteria to be more cost-effective.

In this context, risk stratification of BE patients based on demographic data, clinical information, and diagnostic or predictive biomarkers of disease progression can facilitate more targeted screening, subsequent surveillance, and early-stage treatment, thus increasing survival rates with optimized resources^16^.

The data found in our study suggest that smoking or former smoking, as well as the extent of BE (in centimeters), were risk factors for the development of EAC, similar to findings in the meta-analysis by Krishnamoorthi, which included 74,943 patients^15^. In that meta-analysis, age and male gender were also highlighted as risk factors. However, these variables did not emerge as risk factors in the logistic regression analysis conducted in this study, even though they were statistically significant in the group analysis.

When analyzing patients who developed only dysplasia, age and the extent of BE (in centimeters), were identified as risk factors for its development. As observed in the literature^20^, dysplasia remains the strongest marker for progression to adenocarcinoma, with a 206-fold higher risk compared to patients who did not present dysplasia.

In the analyzed population, BMI did not show a statistically significant difference between the group that developed EAC and the group that did not. However, it is important to note that the average BMI was above 25, suggesting that patients who did not develop EAC were also overweight, complicating the analysis of BMI’s influence on the higher incidence of this outcome^13,25^.

After analyzing the studied population, we can identify the risk factors associated with the development of dysplasia and EAC. By observing the performed endoscopies, it becomes possible to evaluate the maximum follow-up period capable of early detection of EAC and dysplasia. Thus, a critical analysis of the proposals from international societies for EB surveillance against our data becomes essential, especially since there are no guidelines customized for the Brazilian context.

In the present population, non-smoking patients with dysplasia did not develop EAC within 12 months, similar to patients with short-segment BE. In contrast, both smoking patients and patients with long-segment BE developed EAC in less than 12 months. These findings suggest that the follow-up of patients in these specific groups (non-smokers or short-segment BE) could be conducted over a longer period, contrary to the main international recommendations^9^.

This analysis of a small cohort from a single service shows the relevance of associating epidemiological and clinical data (smoking status and extent of BE) with dysplasia (the main risk factor considered in international follow-up guidelines) to optimize and tailor follow-up to the reality of each region, considering cost-effectiveness.

Being a retrospective study, one of its limitations was the inability to evaluate the total sample of 646 patients for all variables, as some information was missing from the records. Continuation of this study, seeking a larger sample size and association with multicentric data, should be encouraged to obtain increasingly reliable data and to propose follow-up guidelines for BE based on the characteristics of our population.

## CONCLUSIONS

The follow-up suggested for our population by international guidelines was appropriate for patients with risk factors for the development of EAC (smoking and long-segment BE). However, patients without risk factors could have their follow-up extended to 12 months, even with a prior diagnosis of dysplasia.
